# Psychosocial support interventions for women with gestational diabetes mellitus: a systematic review

**DOI:** 10.4069/kjwhn.2021.05.13

**Published:** 2021-06-18

**Authors:** Seulgi Jung, Yoojin Kim, Jeongok Park, Miyoung Choi, Sue Kim

**Affiliations:** 1 Graduate School, Yonsei University, Seoul, Korea; 2 National Evidence-based Healthcare Collaborating Agency, Seoul, Korea; 3 Mo-Im Kim Nursing Research Institute, Yonsei Evidence Based Nursing Centre of Korea: a JBI Affiliated Group, College of Nursing, Yonsei University, Seoul, Korea

**Keywords:** Gestational diabetes, Psychosocial support systems, Social support, Systematic review

## Abstract

**Purpose:**

This study aimed to analyze the content and effectiveness of psychosocial support interventions for women with gestational diabetes mellitus (GDM).

**Methods:**

The following databases were searched with no limitation of the time period: Ovid-MEDLINE, Cochrane Library, Ovid-Embase, CINAHL, PsycINFO, NDSL, KoreaMed, RISS, and KISS. Two investigators independently reviewed and selected articles according to the predefined inclusion/exclusion criteria. ROB 2.0 and the RoBANS 2.0 checklist were used to evaluate study quality.

**Results:**

Based on the 14 selected studies, psychosocial support interventions were provided for the purpose of (1) informational support (including GDM and diabetes mellitus information; how to manage diet, exercise, stress, blood glucose, and weight; postpartum management; and prevention of type 2 diabetes mellitus); (2) self-management motivation (setting goals for diet and exercise management, glucose monitoring, and enhancing positive health behaviors); (3) relaxation (practicing breathing and/or meditation); and (4) emotional support (sharing opinions and support). Psychosocial supportive interventions to women with GDM lead to behavioral change, mostly in the form of self-care behavior; they also reduce depression, anxiety and stress, and have an impact on improving self-efficacy. These interventions contribute to lowering physiological parameters such as fasting plasma glucose, glycated hemoglobin, and 2-hour postprandial glucose levels.

**Conclusion:**

Psychosocial supportive interventions can indeed positively affect self-care behaviors, lifestyle changes, and physiological parameters in women with GDM. Nurses can play a pivotal role in integrative management and can streamline the care for women with GDM during pregnancy and following birth, especially through psychosocial support interventions.

## Introduction

Gestational diabetes mellitus (GDM) is the most common medical complication during pregnancy and is defined as diabetes mellitus (DM) or impaired glucose tolerance first detected during pregnancy with the secretion of placental hormones necessary for the fetus to grow [1]. Changes in hormone metabolism, such as estrogen, progesterone, prolactin, and placental hormones [2], as well as increased weight due to increased food intake and reduced levels of physical activity, can result from increased insulin resistance [3].

The prevalence of GDM is reported to be 3% to 14% of pregnant women worldwide and has been steadily increasing [4]. GDM can have a significant impact on obstetric complications and perinatal fetal mortality, and various health problems such as neonatal hypoglycemia, respiratory distress syndrome, obesity, DM, and a decline in brain development after childbirth [5]. A history of GDM increases women’s probability of being diagnosed with GDM in the next pregnancy by 30% to 50% [6], and progression to type 2 diabetes mellitus (T2DM) occurs in 35% to 60% of women [7]. Therefore, strategies to promote self-management are required to prevent complications during pregnancy and childbirth [1].

According to the literature, the effectiveness of intervention programs for women with GDM has been confirmed to a certain extent, especially with regard to physical and dietary interventions. Consuming a low-glycemic index diet and increasing activity levels lowered blood glucose levels, helped reduce insulin requirements during pregnancy, and had a positive effect on appropriate maternal weight gain and reduction in macrosomia rates [8]. In addition, regular moderate-intensity exercise helped control postprandial blood glucose levels in women with GDM [9]. Pregnant women with GDM, however, are confronted with a complex situation where they must acquire knowledge about GDM and practice a healthy lifestyle at the same time as they are diagnosed [10]. Pregnant women diagnosed with GDM are under stress due to the psychosocial changes that pregnancy brings, and concerns that GDM can negatively affect the health of the mother and the fetus can prompt them to feel more stress and depression than women with normal pregnancies, making it difficult to control their blood glucose levels [11]. These concerns can cause women to feel pressured to do well with treatment, which may lead to further stress and anxiety [12]. Postpartum women with GDM need regular self-management such as weight management, diet management, exercise, breastfeeding, and blood glucose testing [13]. However, due to the lack of evidence-based information on the necessity of postpartum care and health beliefs about how these efforts affect health promotion in the future, self-management for GDM has not been found to be effective in most studies [14]. Of particular note, it is difficult for women with GDM to practice self-management due to lack of time and childcare [15].

As such, women with GDM experience various psychological changes and find it difficult to practice self-management due to a lack of social support. Therefore, it is necessary to provide psychosocial support interventions to facilitate regular self-management among women with GDM. Nursing interventions that reflect the needs of pregnant women with GDM and include psychosocial support, taking into account the complex situation of pregnancy and GDM management, have a positive effect on the health of pregnant women and fetuses, as well as prevention of type 2 DM (T2DM). However, intervention studies for women with GDM have mainly focused on diet and exercise, and as such, systematic reviews have been mostly conducted on these topics. Few systematic investigations have focused on the psychological aspects of women with GDM, such as stress, anxiety, or social support. Therefore, this study aimed to analyze the content and impact of psychosocial interventions for women with GDM, and to evaluate their effectiveness. This research is ultimately expected to provide basic data for the development of interventions for GDM education programs.

## Methods

Ethics statement: This study is a systematic review of previously published studies and therefore received an exemption from the Institutional Review Board of Yonsei University Health Systems (Y-2020-0130).

### Study design

The study was conducted and described in accordance with the guidelines for systematic literature review reporting of (Preferred Reporting Items for Systematic Reviews and Meta-Analyses, PRISMA 2020) [16]. The study protocol was registered in the International Prospective Register of Systematic Reviews (PROSPERO) at the National Institute for Health Research (registration number: CRD42020221764).

Following a search of the literature, processes of literature selection, literature quality evaluation, and data extraction were conducted. In order to ensure consistency when selecting literature and evaluating the quality of the literature, two researchers (the main researcher and an assistant researcher with systematic review expertise) independently conducted assessments, and in instances of disagreement, a decision was made through discussion together.

### Search of the literature

Nine online databases were used to search for literature published in domestic and international journals. The following international databases were searched based on the COSI (core, standard, ideal) model [17]: Ovid-MEDLINE, Cochrane Library, Ovid-Embase, Cumulative Index for Nursing Allied Health Literature (CINAHL), and PsycINFO. The domestic databases searched were the National Discovery for Science Leaders (NDSL), KoreaMed, Research Information Sharing Service (RISS), and Korean Studies Information Service System (KISS). Keywords such as “diabetes, gestational,” “psychosocial support systems,” “psychosocial support,” “psychological support,” “social support,” “stress,” “anxiety,” and “depression” were used. A manual search was also conducted to review the references of the selected literature. The databases were searched with no limitation of the time period, and the final search for literature selection was conducted on September 22, 2020 (Supplementary Material).

### Criteria for selection and exclusion of literature

#### Criteria for inclusion of literature

In this study, using the participant, intervention, comparison, outcome, study design (PICO-SD) framework, the following criteria were applied: studies of women with GDM, studies of interventions and programs including psychosocial support, studies in which the effectiveness of an intervention was reported, studies published in English or Korean, and peer-reviewed studies.

• Participants: The participants were pregnant women diagnosed with GDM or women with a GDM history within 5 years of childbirth, who had not been diagnosed with T2DM. GDM is diagnosed in the second or third trimester of pregnancy, by a 75-g oral glucose tolerance test (OGTT) with a one-step approach [18], a 100-g OGTT with a two-step approach [19], or according to the guidelines of the Australian Diabetes in Pregnancy Society (ADIPS) [20] (Appendix 1).

• International Association of Diabetes and Pregnancy Study Group criteria (one-step approach): A 75-g OGTT is performed in a fasting state, and if at least one marker of plasma glucose is abnormal, GDM is diagnosed (fasting plasma glucose [FPG] ≥92 mg/dL, 1-hour plasma glucose ≥180 mg/dL, and 2-hour plasma glucose ≥153 mg/dL).

• Carpenter criteria (two-step approach): Regardless of fasting, a screening test of 50-g OGTT is performed, and if the result is higher than 140 mg/dL, it is determined as positive. If two or more plasma glucose levels are abnormal, GDM is diagnosed (FPG ≥95 mg/dL, 1-hour plasma glucose ≥180 mg/dL, 2-hour plasma glucose ≥155 mg/dL, and 3-hour plasma glucose ≥140 mg/dL).

• ADIPS guidelines: FPG ≥ 5.5 mmol/L or 2-hour plasma glucose ≥ 8.0 mmol/L on a 75-g OGTT.

• Intervention: intervention and education programs, including psychosocial support (informational support, motivational encouragement for self-management, relaxation, emotional support) at least two times or more than 30 minutes.

• Comparisons: usual care or nonintervention that did not provide psychosocial support interventions.

• Outcomes: A classification of the dependent variables of randomized controlled trials (RCTs) and non-RCTs that included psychosocial support interventions for women with GDM through a literature review.

• Behavioral variables: self-management (self-care behavior), practicing healthy eating habits (energy from total fat, fiber intake), and practicing healthy physical activities.

• Psychosocial variables: self-efficacy, prenatal attachment, maternal identity, psychological distress, stress, depression, anxiety, emotional adjustment to diabetes, positive mental health, motivation to change, cues to action, barriers for physical activity and diet, health-related quality of life, social support, perceived susceptibility, perceived severity, perceived benefit, perceived barriers, and risk perception of T2DM.

• Physiological variables: FPG, 1-hour postprandial glucose (PP1hr), 2-hour postprandial glucose (PP2hrs), glycated hemoglobin (HbA1c), 75-g OGTT, glycated albumin, insulin resistance, systolic blood pressure, diastolic blood pressure, triglyceride, low-density lipoprotein, high-density lipoprotein, total cholesterol, body weight after childbirth, body mass index (BMI) after childbirth, waist circumference, and weight loss after childbirth.

• Study design: RCT and non-RCTs were included.

#### Criteria for exclusion of literature

The following studies were excluded from the selection of literature: non-original articles (editorials, reviews, letters and opinion pieces, etc.), gray literature (theses, congress presentation, conference material, abstracts, etc.), studies not focused on women with GDM, studies that did not present an intervention or program that included psychosocial support, qualitative studies, those not reporting the effectiveness of the intervention, and those not published in English or Korean.

### Process of literature selection

In total, 1,801 studies were identified through the aforementioned databases and two more were added through a manual search of the references, finally confirming 1,803 studies. Duplicate literature was eliminated (n=872) through the EndNote X9 program (Clarivate Analytics, Philadelphia, PA, USA) and by hand. Finally, studies were excluded according to the exclusion criteria (n=869).

**Figure 1.**
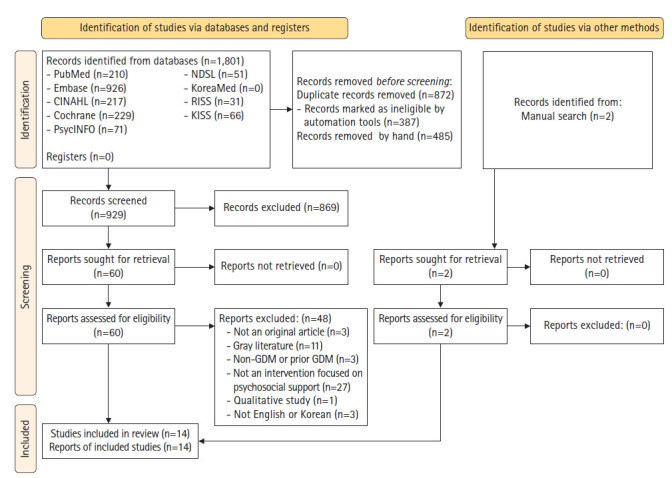
PRISMA 2020 flow chart for the literature search. GDM, Gestational diabetes mellitus.

In the first selection process, the title and abstract were checked to determine whether to select or exclude the document, and for the 60 studies remaining, the secondselection process involved reviewing the full text to determine whether to include or exclude it. After excluding 48 studies, 14 studies were selected (Figure 1).

### Literature quality evaluation

The quality of the final selected articles was evaluated using the Cochrane Risk of Bias (RoB) 2.0 for RCTs [21], and the Health Insurance Review & Assessment Service (HIRA) Study Design Algorithm for Medical Literature of Intervention (DAMI) and the Risk of Bias assessment tool for Non-Randomized Studies (RoBANS) 2.0 for non-RCTs [22].

The RoB 2.0 was used to assess the quality of RCTs in six areas: bias arising from the randomization process, bias due to deviations from intended interventions, bias due to missing outcome data, bias in measurement of the outcome, bias in selection of the reported results, and overall bias. RoBANS 2.0 was applied to non-RCTs to evaluate quality in eight areas: comparability of participants, selection of participants, confounding variables, intervention measurement, blinding of the outcome assessment, outcome evaluation, incomplete outcome data, and selective outcome reporting. In each area of the RoB 2.0 and RoBANS 2.0 tools, the risk of bias was judged as low, high, or unclear. In order to ensure the consistency of the literature quality evaluation, two researchers (the first and second authors) independently conducted it, and in cases of disagreement, consensus was reached through a reevaluation after discussing together.

### Data extraction and synthesis

Data were extracted using a predefined format that included the author’s name, publication year, country of the study, study design, sample, content of the intervention, intervention methods, interventions sessions, measurement time, and outcomes of the intervention (behavioral, psychosocial, and physiological variables). We did not perform a meta-analysis because of heterogeneity in the population and intervention characteristics. We synthesized the results quantitatively.

## Results

### General characteristics of the selected studies

Among the 14 articles selected for the evaluation of the psychosocial intervention program, studies conducted prior to 2013 could not be identified. Eight studies (57.1%) [23-30] were published before 2018, and six studies (42.9%) [31-36] were published after 2018. In the past 3 years, psychosocial support interventions for women with GDM have been actively studied, and it can be confirmed that various intervention methods such as smartphone- or web-based interventions are being used. There were nine international studies and five domestic studies done in Korea (n=5, 35.7%) [28-30,34,36], which was the country with the most studies included in this analysis, followed by Iran (n=4, 28.6%) [24,25,33,35], while one study (7.1%) each was included from Turkey [31], Netherlands [32], United Kingdom [23], Australia [26], and Ireland [27]. The total number of study participants was 1,331, with eight studies (57.1%) [24,25,28,30-33,36] having more than 50 but fewer than 100 participants, three studies (21.4%) [23,26,35] having more than 100 participants, and three studies (21.4%) [27,29,34] having fewer than 50 participants. Ten studies (71.4%) [23-25,28-31,33-35] were conducted among women with GDM during pregnancy and four (28.6%) [26,27,32,36] focused on women with a GDM history within 5 years of childbirth, who had not been diagnosed with T2DM. The studies comprised seven RCTs (50.0%) [23-27,31,32] and seven non-RCTs (50.0%) [28-30,33-36] (Table 1).

### Literature quality evaluation results

**Figure 2.**
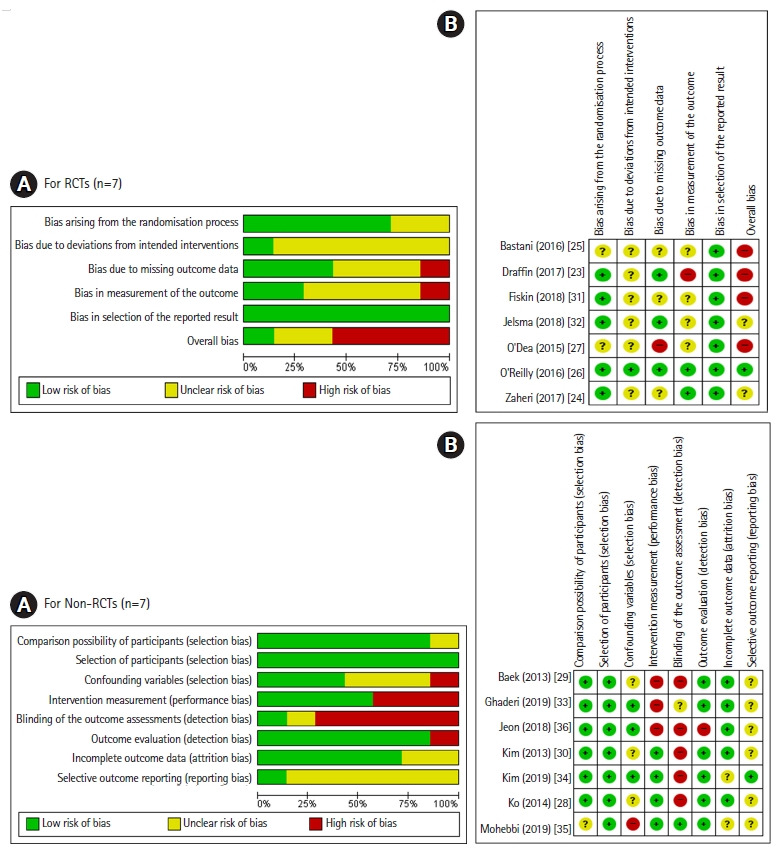
Risk of bias graphs. (A) Risk of bias summary. (B) Risk of bias for selected studies. RCT, randomized controlled trial.

The quality evaluation of the literature showed that five of the seven RCTs (71.4%) [23,24,26,31,32] were well randomized; although the remaining two studies (28.6%) [25,27] were described as involving random assignment, the method was not reported. Six studies (85.6%) [23-25,27,31,32] did not clearly state whether either the participants or researchers were aware of the intervention received by study participants, and only one study (14.3%) [26] was found to be well-blinded for both. As such, bias due to deviations from intended interventions was of some concern. Three studies (42.9%) [23,26,32] used an intent-to-treat (ITT) analysis to correct for bias due to missing outcome data. Although all studies used appropriate methods of measuring the outcome, only two studies (28.6%) [24,26] reported that the outcome assessors were not aware of the intervention received by study participants. This suggests the possibility that outcome assessment may have been influenced by knowledge of the intervention received. All studies (100%) [23-27,31,32] reported outcome data according to a predefined analysis plan. No studies were excluded as a result of the quality assessment. However, the selected literature was assessed overall as being somewhat risky in terms of bias, so the results should be interpreted carefully (Figure 2).

Six of the seven non-RCTs (85.6%) [28-30,33,34,36] confirmed the homogeneity of the experimental group and the control group. All studies (100%) [28-30,33-36] were prospective studies, and three studies (42.9%) [33,34,36] reported a sufficient follow-up period to correct for variables may have been disturbed by the learning effects, and the overall selection bias was evaluated to be low. In four studies (57.1%) [28,30,34,35], the measurements were obtained from reliable sources such as medical records and measured at least two times, so the performance bias was low. One study (14.3%) [35] stated that the evaluator was well-blinded for the outcome assessments. In the outcome evaluation, the results were evaluated well by using tools with proven reliability and validity in six studies (85.6%) [28-30,33-35]. There were two studies (28.6%) [34,35] for which it was difficult to confirm whether incomplete results were presented, and overall the probability of attrition bias was low. One study (14.3%) [34] reported outcome variables such as HbA1c, FPG, and PP1hr, which are expected to be mainly reported in GDM studies, and for six studies (85.6%) [28-30,33,35,36], it was difficult to determine whether selective results were reported. Thus, a possibility of reporting bias was found overall (Figure 2).

### Content of psychosocial support interventions for women with GDM

Among the 10 studies conducted on pregnant women diagnosed with GDM, seven programs [23,28-30,33-35] focused on promoting lifestyle changes (management of diet, exercise, stress, and blood glucose), and other psychological support interventions involved diaphragmatic breathing exercises [31], a stress reduction intervention applying cognitive-behavioral stress management training [24], and an anxiety reduction intervention through acupressure [25] (Table 2).

Four studies were conducted on women with a history of GDM, most of which were diabetes prevention programs [26,27,32,36]. The lifestyle interventions were for women with a history of GDM and BMI more than 25 kg/m^2^ [32], women diagnosed with GDM within the last 1 year [26], women with a history of GDM who had been diagnosed with prediabetes [27], and women who give birth after being diagnosed with GDM [36] (Table 2).

Of the selected articles, 12 used individual interventions (85.7%) [23,25-34,36], six used group interventions (42.9%) [24,26-28,30,35], and four used both types of interventions (28.6%) [26-28,30]. The intervention methods were face-to-face in six studies (42.9%) [25-27,29,31,32], phone-based in six studies (42.9%) [26,28-30,32,36], pamphlet-based in three studies (21.4%) [30,31,36], and video-based in two studies (14.3%) [23,36], while one study each (7.1%) used smartphone-based [33], web-based [34], and short message service and postcard-based interventions [32]. Eight studies (57.1%) [26-32,36] used two or more methods (Table 1).

The total number of sessions was up to 10 in seven studies (50.0%) [23,24,26,29,30,35,36], 10 to 20 in six studies (42.9%) [25,27,28,32-34], and 30 times in one study (7.1%) [31]. The total duration of the interventions was 2 to 4 hours in four studies (28.6%) [30-32,35], more than 4 hours in four studies (28.6%) [24,27,28,34], and 30 minutes to 2 hours in three studies (21.4%) [23,25,36], while three studies (21.4%) [26,29,33] did not specify the total intervention duration (Table 1).

Psychosocial support interventions were found to provide (1) informational support, (2) self-management motivation, (3) relaxation, and (4) emotional support. Among them, informational support was the most common, as it was addressed in 12 studies (85.7%) [23,24,26-30,32-36], followed by 11 studies (78.6%) [23,24,26-30,32-36] that promoted motivation for self-management, and four studies each (28.6%) that used relaxation [25,28,30,31] or emotional support [23,28-30] (Table 3).

In a detailed analysis of the 12 interventions providing informational support, diet management was the most common (n=8, 57.1%) [23,26-28,30,32-34]. Six studies each dealt with GDM information (42.9%) [23,28-30,33,35] and T2DM prevention (42.9%) [23,26,28,30,33,36]. Among the 11 interventions provided for the purpose of self-management motivation, strengthening health behavior practices was the most common (n=11, 78.6%) [23,24,26-30,32,34-36]. Among the four interventions provided to promote relaxation, deep breathing was the most common (n=3, 21.4%) [28,30,31]. Other measures used were acupressure [25], yoga [28], and encouraging *taekyo* (Korean traditional prenatal bonding and interacting with the fetus) [30]. Finally, from the four interventions provided for the purpose of emotional support, sharing opinions and supporting each other in small groups was the most common (n=2, 14.3%) [28,30], while other measures included encouraging expression of positive feelings toward maternal and fetal outcomes [23], willingness to self-manage [28], and emotional status [29] (Table 3).

### Effectiveness of psychosocial support interventions for women with GDM

The effectiveness of the psychosocial support interventions for women with GDM was evaluated by categorizing the outcomes of the interventions conducted in the literature into (1) behavioral variables, (2) psychosocial variables, and (3) physiological variables.

Half of the selected studies (n=7) reported behavioral variables. Of the five studies [28,30,34-36] that analyzed behavioral change, four studies [28,30,34,35] noted increased self-care behavior with statistical significance. Changes in psychosocial variables after psychosocial support intervention were reported in all studies, and depression, anxiety, self-efficacy, and stress were the major variables. Out of the studies dealing with depression (n=7 [24,26-29,31,34]), anxiety (n=7 [23-25,27,29,31,34]), and stress (n=4 [23,24,27,31]), statistically significant improvements were found in four studies [24,28,29,31], five studies [24,25,29,31,34], and three studies [24,27,31], respectively. Of the six studies [23,27,29,32,35,36] dealing with self-efficacy, four studies [29,32,35,36] demonstrated statistically significant increases after the intervention. Finally, for physiological parameters, out of the 14 selected studies, nine studies [23,24,26-28,30,34-36] measured FPG, HbA1c, and PP2hrs as main variables. Out of the studies measuring FPG (n=5 [24,26-28,34]), HbA1c (n=4 [28,30,34,35]), and PP2hrs (n=3 [26,27,30]), statistically significant improvements were reported in three studies [24,26,28], three studies [28,34,35], and two studies [27,30], respectively (Table 4).

## Discussion

This study was conducted to analyze the content and effectiveness of psychosocial interventions for women with GDM. Most of the 14 selected studies were conducted in Korea (35.7%) or Iran (28.6%). Since sociocultural factors are very important factors in the management of GDM [37], psychosocial support interventions should be utilized with active consideration of the sociocultural background of various countries.

The reviewed studies mostly had 50 to 99 participants [24,25,28,30-33,36] and half were of RCT design [23-27,31,32], while there were only four multi-center intervention studies [23,24,26,35]. There is a need for more well designed RCTs in the future, expanding to a greater number of participants, and a need to actively conduct multi-center and multi-national studies.

In terms of study quality, seven RCTs [23-27,31,32] were evaluated as having a low overall level of bias in the randomization process and selection of the reported results, while there was a likely overall risk of bias in the areas of deviations from intended interventions, missing outcome data, and measurement of the outcomes. Only three studies [23,26,32] used an ITT analysis, underscoring the need for more ITT analysis studies to correct for bias due to missing outcome data. In the seven non-RCTs [28-30,33-36], selection bias, performance bias, and attrition bias were evaluated to be low overall, but there was a possibility of detection bias because the evaluators’ blinding was not mentioned [33] or impossible, as researchers provided interventions for participants directly, making it difficult to blind the outcome evaluators. For example, researchers reviewed the participants’ health diaries [34], provided a postnatal care program for GDM postpartum women [36], and helped participants change their health behavior [28]. In two studies involving questionnaires, the researcher conducted a consultation on self-management after questionnaire administration [29] or collected the questionnaire immediately [30]. Furthermore, six studies [28-30,33,35,36] were found to have possible reporting bias. In the future, it is necessary to ensure that the evaluators are well-blinded to minimize the risk of detection bias.

Most of the selected studies (n=10, 71.4%) were aimed at pregnant women diagnosed with GDM, of which the most common focus was on promoting lifestyle changes, whereas only four dealt with women with GDM after childbirth. Because pregnant women diagnosed with GDM are seven times more likely to develop T2DM than pregnant women who maintain normal blood glucose [38], GDM interventions are not only needed for pregnancy, but should be continued after childbirth. It is possible that the lack of GDM intervention studies continuing beyond pregnancy is related to the fact women diagnosed with GDM are often referred to a separate clinician for diabetes management [30], which may result in women with GDM feeling confusion and disconnection in care, as well as decreased collaborative follow-up. Therefore, nurses can play a pivotal role, especially linking clinical departments (internal medicine and obstetrics), and helping women with GDM to continue practicing self-care beyond childbirth.

Individual interventions were performed in most of the studies, and group interventions were used in six studies (42.9%). In addition, various methods such as face-to-face interventions, telephone interventions, pamphlets, and videos were used. One-to-one coaching provides effective knowledge acquisition by enabling participants to receive advice tailored to individual needs and levels [39]. It is effective to organize small-group meetings with 5 to 10 people. If there are more than 10 people, it is difficult to meet individual learning needs [40]. Other studies provided video that stimulated learners’ curiosity and enhanced their understanding and satisfaction [41] or informational support using smartphones, which have advantages such as accessibility and economics [33]. It is necessary to find additional interventions that can provide relaxation and emotional support.

Although half of the studies had fewer than 10 sessions, of the four studies [28,30,34,35] that presented statistically significant results on changes in self-care behavior, it is notable that the frequency of intervention was 4 to 12 times, the total intervention duration was 20 to 60 minutes per session, and the total duration was 2.3 to 6 hours. In GDM management, it is important to provide regular and consistent interventions to facilitate changes in self-care behavior; these four studies appear to suggest that at least four sessions, with more than 20 minutes per session, for a total of 2.3 hours or more is recommendable to promote changes in self-care behavior.

While this review found that informational support and motivational encouragement for self-management were frequently used, relaxation and emotional support were underused. Failure to recognize the seriousness of GDM due to poor education and poor knowledge of health can make it difficult to practice self-management for GDM [42]. On the contrary, regular motivation allows women to adapt well to self-management of GDM [43]. Therefore, informational support for women lacking GDM knowledge, along with strategies for strengthening women’s motivation, is needed and should be continued. In addition, psychosocial support interventions should actively incorporate emotional support and relaxation, especially considering the fear often associated with GDM diagnosis, concerns about the health of the fetus, and anxiety of developing T2DM [44].

The main behavioral variable affected by psychosocial support interventions was change in self-care behaviors. Diagnosis of GDM can be a motivator for healthy behavioral changes and subsequent lifestyle changes [45], so nurses should actively support women with GDM at the time of diagnosis. In addition, because spousal or family support can promote self-management in women with GDM [46], intervention studies based on family support and actively including family members should be conducted.

Depression, anxiety, self-efficacy, and stress were major psychosocial variables found to improve with intervention. Pregnant women with GDM were found to have depressive symptoms that were 3.78 times as severe as those of women with normal pregnancies, and are known to have higher depression and anxiety due to the possibility of complications of GDM [47]. Furthermore, anxiety symptoms significantly increase the risk of DM [48]. In addition, high psychological stress experienced by pregnant women can negatively affect their emotional changes, maternal role, and fetal attachment [49]. Therefore, GDM should be recognized as a high-risk condition and coordinated psychosocial supportive interventions should be offered to reduce depression, anxiety, and stress.

Self-efficacy is an important determinant for self-management and self-control in GDM. As a perception of confidence or a judgment of one’s ability to perform the actions necessary to achieve the desired outcome [50], it plays an important role in adherence to treatment and control of blood glucose [51]. Therefore, psychosocial supportive interventions should also be offered to increase self-efficacy.

The goal of glycemic control during pregnancy is to maintain an FPG of <95 mg/dL, a PP1hr of <140 mg/dL, and a PP2hrs of <120 mg/dL in both pre-GDM and GDM states [52]. The goal of HbA1c control is less than 6.0% to 6.5% in the first trimester of pregnancy and less than 6.0% in the second trimester, but individualized targets must be set with consideration of the risk of hypoglycemia [53]. Fasting hyperglycemia at more than 105 mg/dL in pregnant women with GDM is a risk factor for serious perinatal complications such as intrauterine fetal death, macrosomia, neonatal hypoglycemia, trauma, jaundice, maternal hypertension, preeclampsia, cesarean delivery, and induced delivery [54]. Since postprandial blood glucose during pregnancy has a very strong correlation with the neonatal outcomes of macrosomia [5], it is appropriate to use physiological variables such as FPG, HbA1c, and PP2hrs as the main outcomes. Since 35% to 60% of women diagnosed with GDM develop T2DM [7], it is necessary to determine whether prediabetes or T2DM develops through a 75-g OGTT 6 to 12 weeks after childbirth. If the test results are normal, the woman should have an annual diabetes screening test [4]. Therefore, when designing an intervention program for postpartum women, the 75-g OGTT 6 to 12 weeks after childbirth should be considered as a physiological variable.

Since it is difficult to change self-care behaviors through one-time diabetes education, which is conducted in internal medicine, it is necessary to ensure that integrated psychosocial supportive interventions between internal medicine and obstetrics can be provided on a regular basis, even after childbirth. At a hospital, internal medicine and obstetrics should work together for women with GDM who complain of anxiety about the negative effects of GDM on the fetus (such as macrosomia, hypoglycemia, and respiratory distress syndrome), fear of childbirth, and awareness of the risk of T2DM. Furthermore, the role of professional nurses who systematically manage pregnant women with GDM and promote collaboration between both departments should be emphasized.

The main limitation of this study is that only studies published in Korean or English were selected. Furthermore, since most studies were determined to have a high potential for performance bias, detection bias, and reporting bias, it is necessary to be careful about generalizing the effectiveness of interventions.

Nevertheless, this study is meaningful in that it is the first systematic review study conducted in Korea focusing on psychosocial support interventions for women with GDM; therefore, it can provide basic data for the development of programs for GDM management and T2DM prevention that actively incorporate psychosocial support components.

Based on the results of this study, psychosocial support interventions for GDM should continue to provide informational support and strengthen the motivation to engage in self-care behaviors, and greater use of relaxation and emotional support is needed for women with GDM to maintain positive health behaviors. Psychosocial supportive interventions positively affect self-care behaviors, depression, anxiety, self-efficacy, and stress, as well as improving FPG, HbA1c, and PP2hrs in women with GDM. Therefore, in addition to diet and exercise therapy, which are the main therapeutics for GDM, integrated and comprehensive interventions that include psychosocial dimensions are needed. GDM control and T2DM prevention are needed by continuing to provide GDM interventions including psychosocial support after childbirth. Nurses can bridge the divided care of women with GDM and advanced practice nurses specializing in diabetes care are well poised to provide integrated management starting at pregnancy and extending beyond birth.

## Figures and Tables

**Table 1.** Comparison of studies’ characteristics (N=14)

Variable	Categories		n (%)	References
Publication year	<2018		8 (57.1)	[23-30]
	≥2018		6 (42.9)	[31-36]
Publication country	Domestic	Korea	5 (35.7)	[28-30,34,36]
	International	Iran	4 (28.6)	[24,25,33,35]
		Turkey	1 (7.1)	[31]
		Netherlands	1 (7.1)	[32]
		United Kingdom	1 (7.1)	[23]
		Australia	1 (7.1)	[26]
		Ireland	1 (7.1)	[27]
Participants	<50		3 (21.4)	[27,29,34]
	50–99		8 (57.1)	[24,25,28,30-33,36]
	≥100		3 (21.4)	[23,25,36]
Population	Women with GDM in pregnancy		10 (71.4)	[23-25,28-31,33-35]
	Women with a GDM history within 5 years of childbirth (not diagnosed with T2DM)		4 (28.6)	[26,27,32,36]
Study design	RCT		7 (50.0)	[23-27,31,32]
	Non-RCT		7 (50.0)	[28-30,33-36]
Intervention level	Individual		12 (85.7)	[23,25-34,36]
	Group		6 (42.9)	[24,26-28,30,35]
	Both		4 (28.6)	[26-28,30]
Intervention methods	Face-to-face		6 (42.9)	[25-27,29,31,32]
	Phone		6 (42.9)	[26,28-30,32,36]
	Pamphlets		3 (21.4)	[30,31,36]
	Video		2 (14.3)	[23,36]
	Smartphone-based		1 (7.1)	[33]
	Web-based		1 (7.1)	[34]
	Text and postcards		1 (7.1)	[32]
	Two or more methods		8 (57.1)	[26-32,36]
Total number of sessions	<10 times		7 (50.0)	[23,24,26,29,30,35,36]
	10–20 times		6 (42.9)	[25,27,28,32-34]
	30 times		1 (7.1)	[31]
Intervention duration	30 minutes–2 hours		3 (21.4)	[23,25,36]
	2–4 hours		4 (28.6)	[30-32,35]
	>4 hours		4 (28.6)	[24,27,28,34]
	Not reported		3 (21.4)	[26,29,33]
Psychosocial support interventions	Informational support		12 (85.7)	[23,24,26-30,32-36]
	Self-management motivation		11 (78.6)	[23,24,26-30,32-36]
	Relaxation		4 (28.6)	[25,28,30,31]
	Emotional support		4 (28.6)	[23,28-30]

GDM: Gestational diabetes mellitus; RCT: randomized controlled trial; T2DM, type 2 diabetes mellitus.

**Table 2.** Characteristics of the selected studies (N=14)

First author (year) [reference]	Country	Study design	Sample	Experimental group	Control group	Measurement time
Fiskin (2018) [31]	Turkey	RCT	Pregnant women with GDM (IUP 24–28 wk)	n=30	n=30	Pretest, at 15 days, post-test (3 times)
				Diaphragmatic breathing exercises	Usual care	
				5 minutes every morning (face-to-face, pamphlets)		
				30 days		
Jelsma (2018) [32]	Netherlands	RCT	Women with GDM history (6–48 months after delivery) and BMI ≥25 kg/m^2^	n=29	n=30	Pretest and post-test (2 times)
				Behavioral LSM education	Usual care	
				Counseling: 1 hour (2 face-to-face), 5 telephone, follow-up (5 via text messaging, 4 postcard)		
				5 months		
Draffin (2017) [23]	United Kingdom (multi-center)	RCT	Pregnant women with GDM	n=75	n=67	Pretest, 2 weeks after intervention, 6–8 weeks post-delivery (3 times)
				Educational DVD (46 minutes) on GDM and GDM management (individual): encouraging positive health behaviors and promoting positive feelings	Usual care	
Zaheri (2017) [24]	Iran (two health centers)	RCT	Pregnant women with GDM, stress score >15	n=40	n=40	Pretest, 2 weeks after the last session (2 times)
				Relaxation and cognitive-behavioral techniques: 2 hours×6 times (group)	Usual care	
				3 weeks		
Bastani (2016) [25]	Iran	RCT	Pregnant women with GDM	n=28	n=29	Pretest and post-test (2 times)
				Nurse-provided acupressure at the true point on the forearm: 3 minutes×3 times×2 (individual)	Acupressure at false point	
				2 days		
O’Reilly (2016) [26]	Australia (multi-center)	RCT	Women with a history of GDM within their first postnatal year	n=206	n=228	Pretest, 3 months/12 months after pretest (3 times)
				Individual education on DPP (1 time), group reviewing and longer-term goal setting (5 times), telephone reviewing and longer-term goal setting (2 times)	Usual care	
				3 months		
O’Dea (2015) [27]	Ireland	Mixed methods	Women with a history of GDM in the past 1–3 years and abnormal glucose tolerance	n=16	n=20	Pretest, post-test, 1-year follow-up (3 times)
		RCT		Individualized assessment, 1-hour group exercise, group education seminar, one-to-one motivational interview and individual goal setting with specialist nurse, physiotherapist, or dietician	Usual care (educational pamphlets and routine follow-up)	
				2.5 hours×12 times (face-to-face and group)		
				12 weeks		
Ghaderi (2019) [33]	Iran	Non-RCT	Pregnant women with GDM (IUP 22–32 weeks)	n=44	n=43	Pretest, 6 weeks after the intervention
				Smartphone-based individual education on GDM and management, postpartum management, T2DM prevention	Usual care	(2 times)
				Monitored the number of log-ins and duration of using the application		
Kim (2019) [34]	Korea	Non-RCT	Pregnant women with GDM (IUP 24–28 weeks)	n=22	n=22	Pretest and post-test (2 times)
				Web-based individual program+nutrition session	Usual care	
				Online health diary once a week, logging daily FPG and number of steps taken		
				20–30 minutes×12 times every week		
Mohebbi (2019) [35]	Iran	Non-RCT	Pregnant women with GDM	n=55	n=55	Pretest, 3 months/6 months after intervention (3 times)
	(multi-center)			Education on self-management	Usual care	
				35–40 minutes×4 times (group)		
				1 month		
Jeon (2018) [36]	Korea	Non-RCT	Postpartum	n=30	n=32	Pretest, 12 weeks post-delivery (2 times)
			women with GDM	Education on postpartum GDM and management:	Provide video after the follow-up measurement	
				20 minutes×1 time (pamphlets and video)		
				Telephone follow-up (5 minutes×3 times)		
				6 weeks		
Ko (2014) [28]	Korea	Non-RCT	Pregnant women with GDM (IUP 24 weeks)	n=34	n=34	Pretest and post-test (2 times)
				LSM coaching: 30 minutes×4 times (education), 30 minutes×4 times (small group)	Usual care	
				Individual telephone coaching: 20 minutes×4 times		
				4 weeks		
Baek (2013) [29]	Korea	Non-RCT	Pregnant women with GDM (IUP 24–28 weeks)	n=19	n=18	Pretest and post-test (2 times)
				Case management program	Usual care	
				National standards for DM self-management education and Bandura’s self-efficacy resources (1 face-to-face interview and 5 telephone calls)		
				2 weeks		
Kim (2013) [30]	Korea	Non-RCT	Pregnant women with GDM (IUP 24–30 weeks)	n=28	n=27	Pretest and post-test (2 times)
				Integrated self-management program combining GDM education and pregnancy care (emotional support, *taekyo*, self-management, abdominal breathing, postpartum prevention of T2DM)	Usual care (provide booklet)	
				: 1 hour×3 times (small group and pamphlets)		
				Checking self-management, abdominal breathing, SMBG: 10–15 minutes×2 times (telephone and pamphlets)		
				5 weeks		

DM: Diabetes mellitus; DPP: diabetes prevention program; DVD: digital video disc; FPG: fasting plasma glucose; GDM: gestational diabetes mellitus; IUP: intrauterine pregnancy; LSM: lifestyle modification; RCT: randomized controlled trial; SMBG: self-monitoring blood glucose; T2DM: type 2 diabetes mellitus.

**Table 3.** Content of selected studies (N=14)

Purpose of intervention	Contents	Randomized controlled trial							Non-randomized controlled trial						
		Fiskin (2018) [31]^†^	Jelsma (2018) [32]^†^	Draffin (2017) [23]	Zaheri (2017) [24]	Bastani (2016) [25]	O’Reilly (2016) [26]^†^	O’Dea (2015) [27]^†^	Ghaderi (2019) [33]	Kim (2019) [34]	Mohebbi (2019) [35]^†^;	Jeon (2018) [36]^†^	Ko (2014) [28]^†^	Baek (2013) [29]^†^	Kim (2013) [30]^†^
Informational support (85.7%)	Give information about GDM (42.9%)			v					v		v		v	v	v
	Give information about DM (21.4%)						v		v			v			
	Diet management (57.1%)		v	v			v	v	v	v			v		v
	Exercise management (21.4%)							v	v						v
	Stress management (35.7%)				v		v		v			v			v
	Blood glucose management (21.4%)			v					v						v
	Weight management (21.4%)			v					v						v
	Good sleep hygiene (7.1%)						v								
	Insulin therapy (14.3%)			v					v						
	Postpartum period management (diet, exercise, stress, weight management after delivery, breastfeeding) (35.7%)						v		v			v	v		v
	Prevention of type 2 diabetes (42.9%)			v			v		v			v	v		v
	Explain how to use educational application (14.3%)								v	v					
Motivational encouragement (78.6%)	Set a goal for management (50.0%)		v				v	v		v	v		v	v	
	Checking diet management (28.6%)									v	v		v		v
	Checking exercise management (28.6%)									v	v		v		v
	Checking glucose monitoring (35.7%)									v	v		v	v	v
	Checking weight management (7.1%)									v					
	Checking postpartum GDM management (7.1%)											v			
	Checking stress management (7.1%)														v
	Enhancing positive health behaviors (78.6%)		v	v	v		v	v		v	v	v	v	v	v
Relaxation (28.6%)	Practicing breathing (21.4%)	v											v		v
	*Taekyo* (7.1%)														v
	Practicing yoga (7.1%)												v		
	Nurse-provided acupressure (7.1%)					v									
Emotional support (28.6%)	Sharing opinions and support for each other (14.3%)												v		v
	Encouragement to express willingness to self-manage (7.1%)												v		
	Encouragement to express emotions (7.1%)													v	
	Promoting positive feelings toward outcomes for woman and baby (7.1%)			v											

DM: Diabetes mellitus; GDM: gestational diabetes mellitus.

† Two or more intervention methods used, e.g.: Jelsma (2018) used face-to-face, telephone, short message service (SMS), postcards.

**Table 4.** Outcomes of selected studies (N=14)

First author (year) [reference]	Variable		
	Behavioral	Psychosocial	Physiological
Fiskin (2018) [31]		Prenatal attachment↑^†^, depression↓^†^, anxiety↓^†^, stress↓^†^	
Jelsma (2018) [32]		Barriers for PA and diet ↓^†^	
		Social support for PA and diet ↑^†^	
		Self- efficacy for PA and diet ↑^†^	
Draffin (2017) [23]		1°: anxiety, 2°: prenatal stress, emotional adjustment to diabetes, self-efficacy, GDM knowledge, risk perception	1°: glycemic control (PP1hrs breakfast glucose↓†)
Zaheri (2017) [24]		1°: psychological stress (depression, anxiety, stress) ↓^†^	2°: glycemic control (FPG↓^†^)
Bastani (2016) [25]		Maternal anxiety↓^†^	
O’Reilly (2016) [26]	2°: energy from total fat, fiber intake, moderate-intensity PA	2°: depressive symptoms	1°: [3 months] diabetes risk factor (Wt↓^†^, WC↓^†^, FPG↓^†^) : [12 months] diabetes risk factor (Wt, WC↓†, FPG↓†)
			2°: reduction in body weight, PP2hrs, SBP, DBP, T.chol↓^†^, TG , LDL↓^†^, HDL↓^†^
O’Dea (2015) [27]	2°: PA and diet	2°: mood (positive mental health, psychological distress, depression, anxiety, stress↓^†^), cognition (perceived social support, motivation to change, exercise self-efficacy, diet self-efficacy↑^†^), wellbeing (QOL↑^†^)	1°: glycemic control (FPG)
			2°: glycemic control (PP2hrs↓^†^, IR), lipid profile (TG, HDL, LDL, T.chol), Wt & WC (Wt, BMI, WC)
Ghaderi (2019) [33]		1°: T2DM risk perception ↑^†^	
		2°: T2DM risk perception ↑^†^	
Kim (2019) [34]	Self-care behavior ↑^† ^	Anxiety↓^†^, depression	Glycemic control (HbA1c↓^†^, glycated albumin, FPG, PP1hr)
Mohebbi (2019) [35]		[3 months] Perceived severity↑^†^	
	Self-management↑^†^[3 months]	[6 months] Perceived susceptibility↑^†^, severity↑^†^, barriers↓^†^, benefits↑^†^, self-efficacy↑^†^, cues to action↑^†^	[6 months] Glycemic control (HbA1c↑^†^)
	Self-management↑^†^ [6 months]		
Jeon(2018) [36]	Self-management	Self-efficacy↑^†^	Glycemic control (75-g OGTT )
Ko (2014) [28]	Self-care behavior↑^†^	Depression↓^†^	Glycemic control (FPG↓^†^, HbA1c↓^†^)
Baek (2013) [29]		Self-efficacy↑^†^, depression↓^†^, anxiety ↓^†^	
Kim (2013) [30]	Self-management ↑^†^	Maternal identity↑^†^	Glycemic control (PP2hrs ↓^†^, HbA1c)

1°: Primary outcome; 2°: secondary outcome; BMI: body mass index; DBP: diastolic blood pressure; FPG: fasting plasma glucose; GDM: gestational diabetes mellitus; HbA1c: glycated hemoglobin; HDL: high-density lipoprotein; IR: insulin resistance; LDL: low-density lipoprotein; OGTT: oral glucose tolerance test; PA: physical activity; PP1hr: 1-hour postprandial glucose; PP2hrs: 2-hour postprandial glucose; QOL: quality of life; SBP: systolic blood pressure; T.chol: total cholesterol; T2DM: type 2 diabetes mellitus; TG: triglycerides; WC: waist circumference; Wt: weight.

† Statistically significant.

## Supplementary materials

Further details on supplementary materials are presented online (available at https://doi.org/10.4069/kjwhn.2021.05.13).

## Appendix 1. Details of selected studies (N=14)

**Table:**

First author (year) [reference]	Diagnostic criteria	Theoretical framework
Baek (2013) [29]	Carpenter criteria 100-g glucose tolerance test	Bandura’s (1977) self-efficacy
Bastani (2016) [25]^†^	Not described (personal communications 3 times, but no answer)	Not described
Draffin (2017) [23]	IADPSG criteria 75-g glucose tolerance test	Not described
Fiskin (2018) [31]^†^	Carpenter criteria 100-g glucose tolerance test*	Not described
Ghaderi (2019) [33]^†^	Carpenter/IADPSG criteria* (diagnosis reported in patient file)	Not described
Jelsma (2018) [32]	ADIPS criteria 75-g glucose tolerance test	Rosal’s (2001) patient‐centered counseling model
Jeon (2018) [36]^†^	Carpenter criteria 100-g glucose tolerance test*	Health belief model
Kim (2013) [30]	Carpenter criteria 100-g glucose tolerance test	Cox’s (1982) interaction model of client health behavior
Kim (2019) [34]^†^	Carpenter criteria 100-g glucose tolerance test*	Not described
Ko (2014) [28]	IADPSG criteria 75-g glucose tolerance test	Whitemore’s (2009) goal-reality-options-will (GROW) coaching model
Mohebbi (2019) [35]	No concerns about clinical diagnostic criteria (diagnosed GDM by physicians)	Health belief model
O’Dea (2015) [27]	IADPSG criteria 75-g glucose tolerance test	Not described
O’Reilly (2016) [26]	ADIPS criteria 75-g glucose tolerance test	Not described
Zaheri (2017) [24]^†^	IADPSG criteria 75-g glucose tolerance test*	Antoni’s (2007) cognitive-behavioral stress management

ADIPS: Australian Diabetes in Pregnancy Society; GDM: gestational diabetes mellitus; IADPSG: International Association of Diabetes and Pregnancy Study Group.

† The author was contacted to determine the diagnostic criteria.
