# Supplementary material for: Psychosocial support interventions for women with gestational diabetes mellitus: a systematic review
Source: Korean J Women Health Nurs. 2021 Jun 18;27(2):75–92. doi: 10.4069/kjwhn.2021.05.13 (PMC9334184; doi:10.4069/kjwhn.2021.05.13)
Supplement: Supplementary file 1 [file kjwhn-2021-05-13-suppl.pdf]

Supplementary Material.

| Databases | No. | Search term | Results |
|---|---|---|---|
| **Ovid-MEDLINE (PubMed)** [Mesh] or [TIAB] search | 1 | "Diabetes, Gestational" [Mesh] OR gestational diabetes mellitus [TIAB] OR GDM [TIAB] | 210 |
| | 2 | "Psychosocial Support Systems"[Mesh] OR Psychosocial Support [TIAB] OR Psychological Support [TIAB] OR "Social Support"[Mesh] OR Social Support [TIAB] "Emotions"[Mesh] OR emotional support [TIAB] OR "Stress, Psychological" [Mesh] OR stress [TIAB] OR "Anxiety" [Mesh] OR anxiety [TIAB] OR "Depression"[Mesh] OR depression [TIAB] | |
| | 3 | "Methods"[MeSH] OR Intervention [TIAB] OR "Education"[MeSH] OR Education [TIAB] OR Program [TIAB] OR "Disease Management"[Mesh] OR Management [TIAB] | |
| | Total | 1 AND 2 AND 3 | |
| **Cochrane Library** [MeSH] or [ti, ab, kw] search | 1 | Diabetes, Gestational OR gestational diabetes mellitus OR gestational diabetes OR GDM | 229 |
| | 2 | Psychosocial Support Systems OR psychosocial support OR social support OR psychological support OR emotional support OR Emotions OR Stress, Psychological OR stress OR Anxiety OR Depression | |
| | 3 | Methods OR intervention OR education OR program OR Disease Management OR management | |
| | Total | 1 AND 2 AND 3 | |
| **Ovid- Embase** [Exp] or [ab,ti] search | 1 | pregnancy diabetes mellitus | 926 |
| | 2 | psychosocial care OR social support OR psychological support OR emotional support OR emotion OR stress OR mental stress OR anxiety OR depression | |
| | 3 | intervention OR education OR program OR disease management OR management | |
| | Total | 1 AND 2 AND 3 | |
| **CINAHL** [TI] or [AB] or [SU] search | 1 | Diabetes, Gestational OR gestational diabetes mellitus OR gestational diabetes OR diabetes mellitus in pregnancy OR GDM | 217 |
| | 2 | psychosocial support systems OR psychosocial support OR psychosocial support OR social support OR psychological support OR emotional support emotions OR stress, psychological OR stress OR anxiety OR depression | |
| | 3 | intervention OR program OR education OR disease management OR management | |
| | Total | 1 AND 2 AND 3 | |
| **PsycINFO** [ti]or [ab]or[su] search | 1 | diabetes, gestational OR gestational diabetes mellitus OR GDM | 71 |
| | 2 | Psychosocial Support Systems OR Psychosocial support OR social support OR psychological support OR emotional support OR emotions OR psychological stress OR anxiety OR depression | |
| | 3 | Intervention OR program OR education OR Disease Management OR Management | |
| | Total | 1 AND 2 AND 3 | |

https://doi.org/10.4069/kjwhn.2021.05.13

| Databases | No. | Search term | Results |
|---|---|---|---|
| NDSL [All] search | 1 | 임신성 당뇨 | 3 |
| | 2 | 심리사회적 지지 \| 심리적 지지 \| 사회적 지지 \| 정서적 지지 \| 스트레스 \| 불안 \| 우울 | |
| | 3 | 중재 \| 교육 \| 프로그램 \| 관리 | |
| | 1 | diabetes, gestational \| gestational diabetes mellitus \| GDM | 48 |
| | 2 | psychosocial support systems \| psychosocial support \| social support \| psychological support \| emotional support \| emotions \| stress, psychological \| stress \| anxiety \| depression | |
| | 3 | intervention \| education \| program \| disease management \| management | |
| | Total | 1 AND 2 AND 3 | 51 |
| KoreaMed [All] search | 1 | diabetes, gestational OR gestational diabetes mellitus OR GDM | 0 |
| | 2 | psychosocial support systems OR psychosocial support OR social support OR psychological support OR emotional support OR emotions OR stress, psychological OR stress OR anxiety OR depression | |
| | 3 | intervention OR program OR education OR disease management OR management | |
| | Total | 1 AND 2 AND 3 | |
| RISS [All] search | 1 | 임신성 당뇨 | 20 |
| | 2 | 심리사회적 지지 \| 심리적 지지 \| 사회적 지지 \| 정서적 지지 \| 스트레스 \| 불안 \| 우울 | |
| | 3 | 중재 \| 교육 \| 프로그램 \| 관리 | |
| | 1 | diabetes, gestational \| gestational diabetes mellitus \| GDM | 11 |
| | 2 | psychosocial support systems \| psychosocial support \| social support \| psychological support \| emotional support \| emotions \| stress, psychological \| stress \| anxiety \| depression | |
| | 3 | intervention \| education \| program \| disease management \| management | |
| | Total | 1 AND 2 AND 3 | 31 |
| KISS [All] search | 1 | 임신성 당뇨 | 3 |
| | 2 | 심리사회적 지지 OR 심리적 지지 OR 사회적 지지 OR 정서적 지지 OR 스트레스 OR 불안 OR 우울 | |
| | 1 | diabetes, gestational OR gestational diabetes mellitus | 63 |
| | 2 | psychosocial support systems OR psychosocial support OR social support OR psychological support OR emotional support OR emotions OR stress, psychological OR stress OR anxiety OR depression | |
| | Total | 1 AND 2 | 66 |
